# Pronostic maternel et périnatal de l'éclampsie à l'hôpital de Tombouctou au Mali

**DOI:** 10.11604/pamj.2020.36.175.17976

**Published:** 2020-07-13

**Authors:** Mamadou Ibrahima Kampo, Seydou Sogoba, Djibril Kassogué, Idrissa Konaté, Oumar Ongoiba, Djibril Sissoko, Fatoumata Sow, Youssouf Traoré, Karim Dembélé

**Affiliations:** 1 Hôpital de Tombouctou, Tombouctou, Mali,; 2 Centre hospitalier universitaire Gabriel Touré, Bamako, Mali

**Keywords:** Eclampsie, grossesse, mortalité maternelle, périnatale, Eclampsia, pregnancy, maternal mortality, perinatal

## Abstract

L´éclampsie représente l´une des principales causes de décès maternels dans le monde. Notre objectif était de déterminer le pronostic maternel et périnatal de l´éclampsie à l´hôpital de Tombouctou, Mali. L´étude a été descriptive, rétrospective du 1^er^janvier 2013 au 31 décembre 2017, incluant les cas d´éclampsies survenues au cours de la grossesse ou l´accouchement à l´hôpital de Tombouctou. Nous avons retrouvé 116 cas sur 4951 accouchements soit un taux d´incidence de 2,3%. Il s´agissait essentiellement de femmes de moins de 26 ans (85,3%), primipare (81%), admise en moyenne 8 heures après la première crise. La césarienne était pratiquée dans 77,6% des cas. Le Sulfate de magnésium a été utilisé dans 75% des cas. Les létalités maternelle et périnatale étaient respectivement à 4,3% et 21,5%. Le facteur de mauvais pronostic maternel était un score Glasgow ≤ 8 à l´admission (p: 0,004). Les facteurs de mauvais pronostic périnatal étaient la résidence hors de la ville de Tombouctou (p: 0,000), l´absence de consultation prénatale (p: 0,020) et l´accouchement par voie basse (p: 0,012). Ainsi, l´amélioration du pronostic maternel et périnatal nécessite un suivi correct des grossesses, la réduction des retards dans l´accès à des soins adéquats.

## Abstract (en)

Eclampsia is one of the leading causes of maternal death worldwide. The purpose of our study was to determine maternal and perinatal prognosis of eclampsia at the Timbuktu Hospital in Mali. We conducted a descriptive, retrospective study of patients with eclampsia during pregnancy or delivery at the Timbuktu Hospital from January 1, 2013 to December 31, 2017. Out of 4.951 deliveries, 116 were marked by eclampsia, reflecting an overall rate of 2.3%. These patients were mainly women younger than 26 years (85.3%), primiparous (81%), admitted on average 8 hours after the first crisis. Cesarean section was performed in 77.6% of cases. Magnesium sulphate was used in 75% of cases. Maternal and perinatal mortality accounted for 4.3% and 21.5%, respectively. Poor maternal prognosis factor was Glasgow score ≤ 8 on admission (p: 0.004). Poor perinatal prognosis factors were to be resident outside the city of Timbuktu (p: 0.000), the absence of antenatal consultation (p: 0.020) and vaginal delivery (p: 0.012). Thus, improving maternal and perinatal prognosis requires proper monitoring of pregnancies and reduction of delays in accessing adequate care.

## Introduction

L´éclampsie se définit par la survenue d´une crise convulsive tonico-clonique dans un contexte de pathologie hypertensive de la grossesse [1]. Il s´agit d´une complication grave de la pré-éclampsie, mettant en jeux le pronostic vital maternel et fœtal à court terme. L´éclampsie représente l´une des principales causes de décès maternel dans le monde notamment dans les pays du Sud où son incidence est encore élevée [2]. Dans notre contexte, elle a été la première cause de décès maternel sur la période de 2012 à 2014 dans une étude non publiée, réalisée à l´hôpital de Tombouctou. Ainsi, la réduction de la mortalité maternelle et l´atteinte de l´objectif 3 de développement durable (ODD) passent par une meilleure connaissance épidémio-clinique de l´éclampsie et une évaluation de nos pratiques.

Au Mali, plusieurs travaux ont été réalisés sur l´éclampsie et ont montré que cette pathologie était associée à une létalité mortalité maternelle importante de 5 à 10% [3, 4]. Par contre aucune étude spécifique n´a encore apprécié le pronostic de l´éclampsie à Tombouctou, dans un contexte de ressources humaines et matérielles limitées accentué par la crise sécuritaire dans le Nord du Mali. En effet, dans la région de Tombouctou, le dispositif sanitaire qui faisait déjà face à plusieurs difficultés a été fortement impacté après l´occupation de la ville par des islamistes et bandits armés en 2012.

L´objectif de ce travail était de déterminer le pronostic maternel et périnatal de l´éclampsie à l´hôpital de Tombouctou du 1^er^janvier 2013 au 31 décembre 2017, après la période d´occupation de la région de Tombouctou par les groupes armés.

## Méthodes

L´étude a été rétrospective, descriptive, sur cinq ans, du 1^er^janvier 2013 au 31 décembre 2017. Etaient inclus tous les cas d´éclampsie anté ou per-partum reçus à l´hôpital de Tombouctou (Nord du Mali) pendant la période d´étude. L´éclampsie a été définie par la survenue d´une crise convulsive tonico-clonique dans un contexte de pathologie hypertensive de la grossesse, non expliquée par une autre cause neurologique. Les cas d´éclampsie ont été colligés à partir des registres (d´admission, d´accouchement et de compte rendu opératoire) et des dossiers médicaux des services de gynécologie obstétrique et des urgences-réanimations.

Les caractéristiques sociodémographiques étudiées étaient l´âge, le sexe, la profession, le statut matrimonial la résidence. Les données cliniques recueillies comprenaient, la parité, le nombre de consultation prénatale (CPN), le délai entre la première crise d´éclampsie et l´admission, le moyen de transport utilisé ainsi que le score de Glasgow et la tension artérielle à l´admission. Le moment de survenue l´éclampsie (ante-partum, per-partum); le nombre de crise, l´âge gestationnel, le délai entre l´admission et l´accouchement ainsi que la voie d´accouchement ont également été recueillis. Le bilan biologique réalisé (Taux d´hémoglobine, plaquettes, ASAT, ALAT, urée, créatininémie, protéinurie), le traitement reçu, la durée d´hospitalisation et l´évolution (favorable, décès, autre complications) ont également été appréciés. Les paramètres du nouveau-né concernaient le poids, la vitalité, le score d´Apgar à la naissance et dans le cas échéant le moment du décès.

Les données ont été saisies sur logiciel Access version 2007 et analysées sur le logiciel Stata version 14.0. Les variables catégorielles ont été analysées avec un test de Chi^2^ exact de Fisher et les variables continues par un test de Student avec un seuil de significativité p = 0,05. L´analyse des données a consisté en une étude descriptive simple des caractéristiques sociodémographiques, cliniques et pronostiques de l´éclampsie parmi les cas colligés. Puis une analyse uni-variée a été faite afin de déterminer les facteurs associés à la létalité maternelle et périnatale par éclampsie.

## Résultats

Durant la période d´étude de 2013 à 2017, nous avons colligé 116 cas d´éclampsie survenues en ante et per-partum sur un nombre total de 4951 accouchements soit un taux d´incidence cumulée de 2,3%. La première crise d´éclampsie était survenue en ante-partum dans 88 cas (75,9%), en per-partum dans 28 cas (24,1%).

**Les caractéristiques sociodémographiques:** l ´âge médian était de 18 ans [IQR= 17-20] avec des extrêmes de 15 et 40 ans. Malgré le jeune âge de nos patientes, il s´agissait de femmes mariées dans 84% cas, leur occupation essentiellement était celle de femmes au foyer. Elles étaient primipares dans 81% des cas et résidaient essentiellement la commune (ville) de Tombouctou (Tableau 1).

**Tableau 1:** caractéristiques des patientes présentant une éclampsie anté ou per-partum l´hôpital de Tombouctou de 2013-2017 (N = 116)

Caractéristiques	Effectif	Pourcentage (%)
**Age (Ans)**		
15-25	99	85,3
26-35	13	11,2
≥ 36	3	2,6
Non précisé	1	0,9
**Parité**		
Primipare	94	81
Paucipare (2-3)	5	4,3
Multipare (≥ 4)	10	8,7
Non précisé	7	6
**Résidence**		
Commune de Tombouctou	78	67,2
Autres	38	32,8
**Age gestationnel**		
≤ 32 SA (semaines d’aménorrhée)	23	19,8
33-36 SA	17	14,6
≥ 37 SA	66	57
Non précisé	10	8,6

**Les données cliniques et thérapeutiques:** concernant le suivi de la grossesse, 36,2% n´avaient réalisés aucune consultation prénatale. Pour le mode d´admission, dans 65% des cas nos patientes étaient amenées par leurs familles. Pour les patientes référées, elles venaient essentiellement d´un centre de santé communautaire (65%) et d´un centre de santé de référence (28%) des cas. La qualification de l´agent qui referait la patiente était une infirmière obstétricienne (50%), un médecin généraliste (27,5%), une matrone (12,5%) et une sage-femme (7,5%). Les moyens de transport utilisés pour arriver à l´hôpital étaient non médicalisés dans 65% des cas. De plus, les patientes ont été admises dans un contexte de retard avec un délai entre la première crise et l´admission en moyenne de 8 heures. Ainsi, la majorité de nos patientes admises pour éclampsie avaient une altération de la conscience à l´admission avec un score de Glasgow entre 9 et 13 dans 70% des cas et ≤ 8 dans 12,9% des cas. Pour la tension artérielle à l´admission, la médiane de la systolique était de 160 mmHg et celle de la diastolique de 100 mmHg. La protéinurie mesurée de manière semi-quantitative à la bandelette urinaire était très significative et ≥ 100 mg/dl (soit ≥ 2 croix) dans 75% des cas. Chez nos patientes le délai médian entre l´admission et l´accouchement était de 2 heures et 90% des patientes ont accouchés dans les 7 heures après l´admission. Le taux de césarienne était de 77,6%. L´indication principale des césariennes était l´état de mal éclamptique. L´accouchement était par voie basse dans 16,24% dont 5,2% d´extractions instrumentales. Nous avons utilisé le Sulfate de Magnésium chez 75% de nos patientes et du Diazépam chez 11,5%.

### Pronostic maternel et périnatal

**Le pronostic maternel:** il est sévère avec un taux de létalité maternelle de 5/116 soit 4,3%. Nous avons retrouvé comme facteur associé significativement à la létalité maternelle par éclampsie, un score Glasgow ≤ 8 à l´admission (Tableau 2). La morbidité associée à l´éclampsie était également importantes. La prévalence de l´anémie était élevée dans notre étude, environ la moitié de nos patientes avaient une anémie (47,4%). Le taux d´hémoglobine moyen était de 10,5 avec un écart-type à 2,5. Les complications associées, liées à l´hypertension gravidique étaient deux cas de HELLP Syndrome, un cas d´hématome rétroplacentaire et un cas d´œdème aigu pulmonaire. Quatre suppurations pariétales ont été observées sur 90 césariennes soit 4,4%. Enfin une éviscération post-césarienne et une déchirure complète compliquée périnée du périnée post-extraction instrumentale ont été retrouvées. La durée moyenne de l´hospitalisation était de 6 jours avec un écart type de 4 jours.

**Tableau 2:** facteurs associés à la létalité maternelle dans les éclampsies anté ou per-partum à l´hôpital de Tombouctou de 2013 à 2017

Facteurs étudiés	Effectif (Prévalence %)	Nombre décès maternel (Létalité %)	p-value
Score Glasgow ≤ 8 à l´admission	15(12,9)	3(20)	**0,004**
Résidence hors commune (ville) de Tombouctou	38(32,7)	3(7,9)	0,185
Parité ≤ 2	98(84,4)	4(4)	0,495
PAD ≥110 mmHg	40(34,5)	2(5)	0,703

**Le pronostic périnatal:** dans la majorité des cas (57%), il s´agissait d´une grossesse à terme avec un poids de naissance moyen était de 2496,5g (SD: 724,2; Min: 500; Max: 4000). Avec 22 cas de mort-né, le taux de mortinaissance était 19 %. Nous avons par la suite déploré trois décès néonatals précoces. Ainsi, le taux de létalité périnatale était élevé dans notre étude avec 25 décès sur 116 soit 21,5 %. Les trois facteurs associés à la létalité périnatale étaient la résidence hors de la commune (ville) de Tombouctou, l´accouchement voie basse et l´absence de consultation prénatale (Tableau 3).

**Tableau 3:** facteurs associés à la létalité périnatale dans les éclampsies anté ou per-partum à l´hôpital de Tombouctou de 2013 à 2017

Facteurs étudiés	Effectif (Prévalence %)	Nombre décès maternel (Létalité %)	p-value
Résidence hors commune (ville) de Tombouctou	33(28,4)	17(51,5)	**0,000**
Accouchement voie basse	19(16,4)	9(47,3)	**0,012**
0 CPN	42(36,2)	14(33,3)	**0,020**
Parité ≤ 2	94(81)	21(22,3)	0,248
Score Glasgow ≤ 8 à l´admission	14(12)	5(35,7)	0,334
PAD ≥110 mmHg	32(27,6)	9(28,1)	0,492
Age gestationnel < 37 SA	40(34,4)	8(20)	0,723

## Discussion

Notre étude des éclampsies sur cinq ans à l´hôpital de Tombouctou, nous a permis de rapporter les premières données sur le sujet dans cette région du Mali avec 116 cas d´éclampsie recensés (2,3% des accouchements). Au Mali peu d´études existent sur la fréquence de l´éclampsie. Ainsi à Gao une étude sur une année trouve une incidence de 5,03% [4]. Deux études réalisées au CHU du Point G à Bamako ont retrouvé que l´éclampsie représentait 6,82 des admissions en période gravido-puerpérale et 22,5% des admissions en service de réanimation [3]. Dans le reste de l´Afrique, notre incidence à 2,3% était supérieure à celles rapportées à Dakar (8 p 1000) et au Nigeria (1,12%) [5, 6]. Elle était cependant moins élevée que les 3,3% rapportés à Ouagadougou [7]. Dans les pays développés par contre, l´incidence de l´éclampsie reste très faible 0,5 à 0,7 p.1000 accouchement [8-11]. Ce constat illustre bien le fait que l´incidence de l´éclampsie peut être réduite avec un meilleur suivi des grossesses et une prise en charge adéquate des hypertensions artérielles gravidiques.

Les caractéristiques sociodémographiques étaient marquées par des sujets majoritairement jeunes, mariées et non scolarisées. Cela pose le problème des mariages précoces encore trop fréquents dans notre contexte et des besoins non satisfaits en contraception. Ces constats sont à l´image de ceux retrouvés dans la cinquième Enquête Démographique et de Santé au Mali EDSM V avec 67% des femmes qui n´avaient aucune éducation et 85% des femmes de 15-49 en unions. Dans ce contexte, l´Etat et ses partenaires mènent à Tombouctou plusieurs actions de sensibilisation et de formation en réponse aux problèmes de mariage précoce, de violences basées sur le genre. Un accent particulier est également mis sur l´offre de service de planification familiale et les spécificités en santé de la reproduction des adolescentes. Concernant le délai entre la survenue de la crise d´éclampsie et l´admission à l´hôpital, elle met en exergue l´ampleur du 2^e^retard puisque le délai moyen était de 8 heures. Nos patientes étaient amenées le plus souvent par leurs parents avec des moyens de transport non médicalisés. Par ailleurs, la région de Tombouctou est caractérisée par un vaste territoire et l´hôpital de Tombouctou est le seul centre de santé de 2^e^référence de la région. Les distances qui la séparent des centres de santé communautaires et des centres de santé de référence peuvent parfois être très importantes. De plus le mauvais état des routes, l´insuffisance des moyens de transport et le contexte d´insécurité constituent autant de défis à relever. Ces difficultés pourraient également expliquer le faible effectif venant des autres cercles de la région. Ainsi, l´amélioration du système de référence-évacuation et la stabilisation de la région sont nécessaires pour assurer un acheminement rapide des patientes vers l´hôpital.

Dans notre série, il s´agissait souvent de grossesses non suivies. De plus, même lorsque les CPN étaient réalisées elles étaient souvent en nombre insuffisant et de qualité sub-optimale. En effet la prise de la tension ou la réalisation de l´albuminurie pourtant essentielles pouvaient faire défaut. Il est donc ici essentiel d´assurer une prise en charge par un personnel qualifié et selon les recommandations nationales. La faible qualification du personnel dans notre contexte se percevait à travers les agents qui ont référés les patientes puisqu´il s´agissait d´infirmières obstétriciennes dans plus de la moitié des cas. Enfin dans environ un tiers des cas il s´agissait de médecins généralistes et de sages-femmes. Cette insuffisance en ressource humaine qualifiée est en grande partie liée au départ massif des agents de santé au moment de l´occupation des régions nord avec peu de retour après la reconquête. Encore de nos jours il n´y a aucun gynécologue-obstétricien dans la région en dehors de l´hôpital de Tombouctou. Ces difficultés en amont de l´admission ont eu pour conséquence un retard de prise en charge avec un taux important d´altération de la conscience chez nos patientes. Dans ce contexte, la prise en charge obstétricale de nos patientes a été le plus souvent rapide puisque 90% avaient accouché dans les 7 heures après l´admission et le Sulfate de Magnésium a été utilisé dans 75% des cas. En effet, l´éclampsie est une complication grave où il est recommandé un traitement par Sulfate de Magnésium et un accouchement dans un bref délai [12]. Concernant la voie d´accouchement, 77,6% des cas d´éclampsie pendant la grossesse et l´accouchement ont eu une césarienne d´urgence. Ce taux est supérieur à ceux que nous avons retrouvé dans la littérature entre 10 et 50% [5,6,13]. Notre taux élevé pourrait s´expliquer par l´état clinique de nos patientes à l´admission avec souvent un état de mal éclamptique dans un contexte de retard à l´admission laissant peu de marge de temps d´intervention. Ainsi, la réduction du deuxième retard a été possible grâce aux efforts de l´Etat et ses partenaires avec la gratuité de la césarienne et la disponibilité du Sulfate de Magnésium. Dans notre étude, le pronostic de l´éclampsie était sévère avec des létalités maternelle et périnatale respectivement à 4,3% et 21,5%. En effet l´éclampsie représente une des principales causes de décès maternel dans le monde [14]. La létalité maternelle observée dans notre série était supérieure à celle observée au Nigeria (2,5%) et dans les pays développés [8, 11, 15]. Elle était toutefois moins importante que celle observée au Burkina Faso (6,4%) les 17,9% au CHU de Dakar (Sénégal) [5, 7].

Les autres facteurs étaient score Glasgow ≤ 8 à l´admission et l´accouchement par voie basse. Cissé *et al*. ont également retrouvé l´accouchement par voie basse comme facteurs associés à la mortalité maternelle ainsi que le nombre de crise supérieur à deux [5]. Nous n´avons cependant pas pu apprécier ce dernier car insuffisamment précisé dans les dossiers. La morbidité maternelle associée était l´anémie, essentiellement en rapport avec le faible suivi des grossesses et l´absence de prophylaxie anti-palustre et de supplémentation en fer. Certaines complications étaient toutefois liées à l´accouchement par césarienne (suppuration pariétale et éviscération) ou l´extraction instrumentale (déchirure complète et compliquée du périnée). Cela rappelle les efforts à faire pour la prise en charge, notamment en matière de prévention de l´infection. Quant à la létalité périnatale à 21,5% dans notre série, elle était plus élevée que celle du CHU point G (Bamako) à 10,18% [3]. Notre taux était inférieur celle rapporté au CHU de Dakar 35,9% [5] et à Ouagadougou 31,5% [7]. Les facteurs associés à cette létalité périnatale dans notre étude étaient la résidence hors de la commune (ville) de Tombouctou, l´accouchement voie basse et l´absence de consultation prénatale. Ainsi pour la réduction de ce taux de létalité périnatale, il est nécessaire d´assurer un suivi prénatal correct des grossesses et de lutter contre les retards d´accès aux soins et de prise en charge.

## Conclusion

L´éclampsie constitue un problème important de santé publique dans notre contexte survenant chez des femmes jeunes. La réduction de la létalité maternelle et périnatale nécessite un meilleur suivi des grossesses, la lutte contre le retard d´accès aux soins et l´amélioration de la prise en charge rapide grâce à un personnel qualifié ainsi que la mise en place d´une couverture sanitaire universelle comme recommandée par l´Organisation mondiale de la santé.

### Etat des connaissances sur le sujet

- L´incidence, les létalités maternelle et périnatale de l´éclampsie sont élevées dans les pays du Sud;
- La majorité des décès dus à l´éclampsie sont évitables si les femmes qui en sont atteintes reçoivent des soins efficaces en temps opportun;
- Le sulfate de magnésium est l´anticonvulsivant de référence pour le traitement de l´éclampsie.

### Contribution de notre étude à la connaissance

- L´incidence, les létalités maternelle et périnatale de l´éclampsie à Tombouctou (Mali);
- Dans notre contexte le retard à l´admission des patientes éclamptiques est si important que malgré le bref délai entre l´admission et l´accouchement, ainsi que le taux de césarienne élevé les létalités maternelle et périnatale sont élevées.
